# Full-Body Photobiomodulation Therapy Is Associated with Reduced Sleep Durations and Augmented Cardiorespiratory Indicators of Recovery

**DOI:** 10.3390/sports10080119

**Published:** 2022-07-31

**Authors:** Lauren E. Rentz, Randy W. Bryner, Jad Ramadan, Ali Rezai, Scott M. Galster

**Affiliations:** 1 Division of Exercise Physiology, West Virginia University School of Medicine, Morgantown, WV 26506, USA; rbryner@hsc.wvu.edu; 2 Rockefeller Neuroscience Institute, West Virginia University, Morgantown, WV 26506, USA; jramadan@hsc.wvu.edu (J.R.); ali.rezai@hsc.wvu.edu (A.R.); scottgalster@gmail.com (S.M.G.)

**Keywords:** athlete monitoring, exercise training, red light therapy, soccer, wearable device

## Abstract

Research is emerging on the use of Photobiomodulation therapy (PBMT) and its potential for augmenting human performance, however, relatively little research exists utilizing full-body administration methods. As such, further research supporting the efficacy of whole-body applications of PBMT for behavioral and physiological modifications in applicable, real-world settings are warranted. The purpose of this analysis was to observe cardiorespiratory and sleep patterns surrounding the use of full-body PBMT in an elite cohort of female soccer players. Members of a women’s soccer team in a “Power 5 conference” of the National Collegiate Athletic Association (NCAA) were observed across one competitive season while wearing an OURA Ring nightly and a global positioning system (GPS) sensor during training. Within-subject comparisons of cardiorespiratory physiology, sleep duration, and sleep composition were evaluated the night before and after PBMT sessions completed as a standard of care for team recovery. Compared to pre-intervention, mean heart rate (HR) was significantly lower the night after a PBMT session (*p* = 0.0055). Sleep durations were also reduced following PBMT, with total sleep time (TST) averaging 40 min less the night after a session (*p* = 0.0006), as well as significant reductions in light sleep (*p* = 0.0307) and rapid eye movement (REM) sleep durations (*p* = 0.0019). Sleep durations were still lower following PBMT, even when controlling for daily and accumulated training loads. Enhanced cardiorespiratory indicators of recovery following PBMT, despite significant reductions in sleep duration, suggest that it may be an effective modality for maintaining adequate recovery from the high stress loads experienced by elite athletes.

## 1. Introduction

Sports at the collegiate, semi-professional, and professional levels each amount to multi-billion-dollar industries in the United States [1]. At elite levels of play, extensive focus is garnered towards athlete recovery to maximize performance and minimize the risk of injury. Undoubtedly, a substantial degree of physiological and mental stress is exerted on the body during highly competitive and physical sports, such as soccer. Recent considerations have also shed light on the additional cognitive and emotional loads that sports can exert on an athlete and how this may interplay with physical demands to ultimately affect performance [2]. The common understanding that sport performance is multi-dimensional should emphasize the concept that recovery should also be multi-dimensional. 

A developing modality for augmented recovery and performance enhancement is Photobiomodulation Therapy (PBMT). This therapy, often termed red light therapy, utilizes specific wavelengths between 620–1100 nm, or combinations thereof, ranging from visible red light up to infrared light on the spectrum [3]. Many theories have been proposed as to the potential cellular mechanisms of PBMT; however, nearly all suggest a strong relationship with enhanced adenosine triphosphate (ATP) production from the mitochondria and cell proliferation [4,5,6,7,8,9,10]. These theories are supportive of recovery at the cellular level, which may translate to tissue or systematic-wide relief from oxidative stress [7,11].

Previous research is suggestive of performance enhancements in athletes following PBMT [12,13,14,15,16], as is recovery from high-intensity exercise, which has most commonly been quantified via blood biomarkers [17]. Despite some auspicious results, other studies refute these claims, with evidence that suggests little to no impact on recovery [18,19]. Until the recent development of full-body beds, PBMT has primarily been administered through localized laser treatments to a target-specific tissue or bodily region. Newer technologies utilizing light emitting diodes (LEDs) have aided in providing treatment to larger anatomical areas. PBMT applications utilizing LEDs and lasers differ in that LEDs have a wider bandwidth and emits light in an incoherent fashion [20]. Presently, much of the existing research regarding PBMT has utilized lasers, though numerous clinical studies have reported a comparable [21,22] or greater effects from LED applications [23,24,25]. Variation among delivery methods and anatomical location, in combination with a wide range of applied wavelengths and dosages, contribute to substantial variation in methodology and findings among extant literature [15,26,27,28]. 

Much of the existing research assessing PBMT on human subjects has been conducted through strict laboratory controlled clinical trials, often obtaining relatively low sample sizes [12,13,18,26,29,30,31,32]. While many of these studies carefully controlled for administration of treatment conditions and the quantification of effects, few studies have assessed free-living use of PBMT while accounting for the inter-individual loads that are accumulated outside of the controlled laboratory setting. As such, strong evidence attesting to the efficacy of implementing PBMT for enhancement of athlete performance and recovery is still warranted.

Autonomic status and balance have been identified as valuable indicators of fatigue and recovery [33,34,35,36,37]. With external influences minimized during sleep, nocturnal monitoring of heart rate (HR) and heart rate variability (HRV) during rest are said to exhibit the strongest degree of reliability for quantifying recovery [37,38], specifically in athletes [39,40,41]. Nocturnal trends in HR and HRV can be influenced by a multitude of factors impacting recovery status, including physical training and sleep quality [41,42,43,44,45]. Despite this, few studies have evaluated sleep or nocturnal physiology in response to PBMT; though, limited research has suggested improvements in subjective sleep quality and serum melatonin [30] despite no effect on next morning HRV [32]. 

The present analysis was conducted to observe natural, free-living trends in sleep and recovery surrounding the use of PBMT in a team of Division I NCAA women’s soccer athletes. Given the intense physical workloads demanded of athletes at this level, in combination with the mental and emotional stress of intra- and inter-team competition, full day-to-day recovery is of the utmost importance for this population to prevent injury or overreaching [46,47]. It was hypothesized that PBMT would be associated with improved physiological indicators of recovery.

## 2. Materials and Methods

### 2.1. Study Procedures

Data were analyzed from a NCAA Division I collegiate women’s soccer team following one competitive season. Only data collected from active, non-injured athletes during in-season training were included. All participants provided their written informed consent to retrospectively share personal data collected by the team. Study procedures were approved by the West Virginia University Institutional Review Board, and all study procedures are in accordance with the Declaration of Helsinki guidelines. 

As a standard practice for team recovery, athletes were provided the opportunity to complete 20-min PBMT sessions using a NovoTHOR full-body light bed (THOR Photomedicine Ltd., Chesham, United Kingdom) emitting visible red (660 nm) and near infra-red (NIR, 850 nm) light. Dose and beam parameters for each session completed with the NovoTHOR bed are provided in Table 1 [48]. Whole-body doses were applied to exposed skin without the hinderance of clothing. As per the team standards, athletes were allowed to complete up to two sessions per week and were not permitted to complete sessions on subsequent days. 

Each athlete wore a properly sized second-generation OURA ring (OURA Health, Oulu, Finland) each night on a consistent finger (2nd, 3rd, or 4th digit) of the dominant hand, which captured sleep and physiological parameters. Athletes were also monitored during all training periods with a Catapult Vector S7 (Catapult Sports, Melbourne, Australia) GPS tracker to capture daily training and competitive physical loads.

### 2.2. Obtained Samples

Twelve athletes, all of which were field players, completed at least one PBMT session throughout the season, totaling 106 sessions. Fourteen sessions were excluded from analysis due to failure to wear the OURA ring either the night immediately before or after the PBMT session. Of the 93 sessions included (average 8.5 ± 7.5 sessions/player), 37 sessions were completed on rest days, in which no physical training took place, and 56 were completed on training days. None of the participating individuals had a diagnosed sleep disorder.

### 2.3. Measures

#### 2.3.1. Cardiorespiratory Physiology

Summary physiological variables, which are representative of the full night and collected via the OURA ring, were obtained for analysis including average HR, average HRV (RMSSD), and average respiration rate (RR). Additionally, five-minute epochs of average HR and HRV were further analyzed throughout each half of the night for intra-night trends. Given that the first and second halves of the sleep phase traditionally differ in NREM and REM propensity, cardiac parameters were evaluated for each half independently, in addition to full night averages. Maximum, average, and average rate of change/hour for HR and HRV, as well as minimum HR, were calculated individually for the first and second half of each sleep period.

#### 2.3.2. Sleep

Summary sleep variables, which are representative of the full night, were collected via the OURA ring and included total sleep time (TST), sleep efficiency (SE), awake duration, light sleep duration, deep sleep duration, and REM sleep duration. Proportions of each sleep stage were further calculated and are included as % Light, % Deep, and % REM.

#### 2.3.3. External Training Load

Training load data collected during training and competitive sessions via Catapult sensors were quantified using player load, which is the tri-axial sum of acceleration during a session. Player loads were compiled as the total player load recorded each day, as well as a cumulative four-day total that included summated values from the day of record and three days prior.

### 2.4. Statistical Analyses

All statistical analyses were conducted in JMP Pro 14 (SAS Institute, Cary, NC, USA). Full night pre-intervention and post-intervention physiological and sleep variables were checked for unequal variance using Bartlett’s test and were determined to have equal variance. Two-tailed paired *t*-tests were used to identify within-individual differences in pre-intervention (night before a session) and post-intervention (night after a session) measures (variable indicated as night). Cohen’s d was utilized to calculate the standardized differences between the paired comparisons.

#### 2.4.1. Intra-Night Relationships

To identify the relationship that sleep duration may have with physiological indicators of recovery, linear mixed models were run on pre-intervention and post-intervention data using a residual structure; three fixed effects were explored for their impact on each of the cardiorespiratory variables including a main effect (ME) of night, ME of TST, and the interaction between night*TST.

Evaluation of HR and HRV variables by half of the night were checked using Bartlett’s test and were determined to have unequal variance; thus, two-way repeated measure Analysis of Variance (ANOVA) assessing within participant fixed effects of night and half of night on HR and HRV were run using a mixed model with an unequal variance structure. All two-way ANOVAs were modeled with three fixed effects (two ME and an interaction of the two). Tukey’s HSD was used to correct for multiple comparisons and identify any resultant pairwise differences.

#### 2.4.2. External Training Load

Linear mixed effects were modeled based upon single-day (designated as 1D player loads) and cumulative four-day player loads (designated as 4D player loads) to identify effects of varying training loads on physiological and sleep parameters.

## 3. Results

Nocturnal physiology and sleep parameters comparing pre-intervention measures to post-intervention measures are listed in Table 2. Of the nocturnal cardiorespiratory measures assessed, only average HR was found to be significantly lower after PBMT as compared to pre-intervention. With regard to sleep, TST, light sleep duration, REM duration, and REM proportion were significantly lower the night after a PBMT session as compared to pre-intervention. 

### 3.1. Intra-Night Relationships

**Figure 1:**
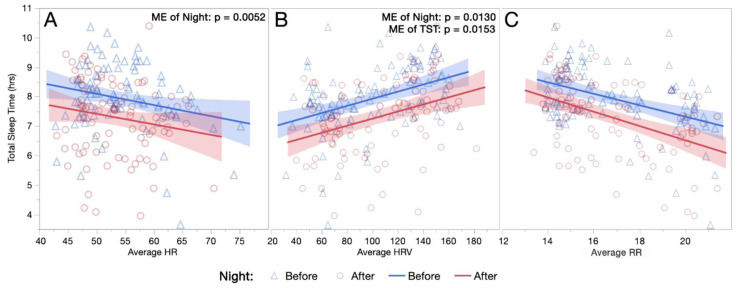
(**A**–**C**) Relationships between Total Sleep Durations and Nocturnal Physiology. The relationship between total sleep duration and nocturnal physiological parameters are demonstrated for both the night before and the night after a PBMT session, including (**A**) average HR, (**B**) average HRV, and (**C**) average RR. (HR, heart rate; hrs, hours; HRV, heart rate variability; ME, main effect; RR, respiration rate).

Figure 1A–C demonstrates the relationship between average physiological parameters and TST by night. All fixed effects from the linear mixed model analyses demonstrating the relationships between these variables are listed in Table 3; despite a significant reduction of almost 40 min in post-intervention TST, significant ME of night existed on average HR and average HRV. 

**Figure 2:**
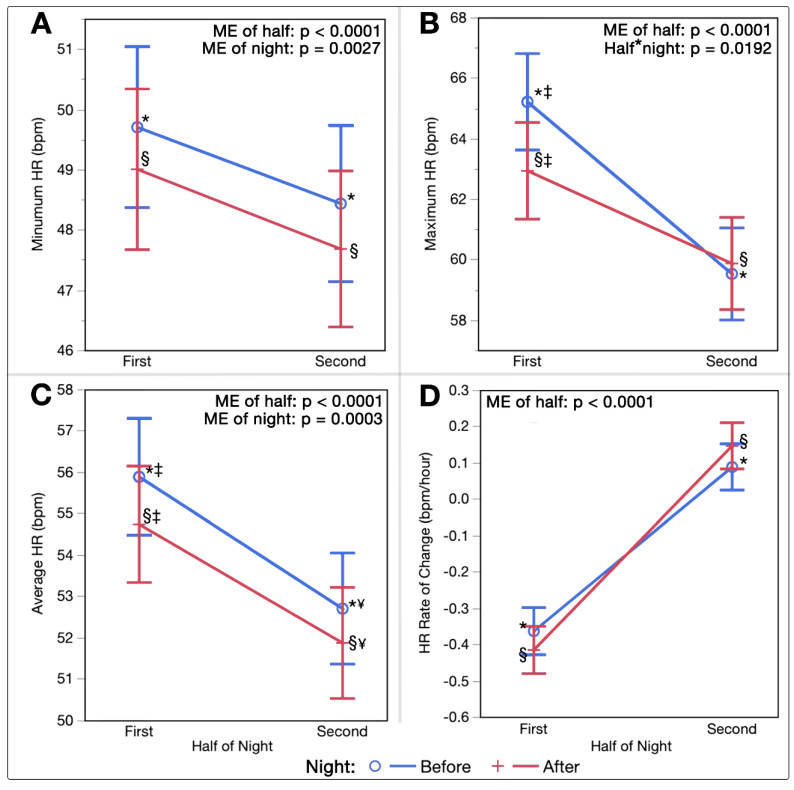
(**A**–**D**) Heart Rate Trends by Half of Night. Least squares means interaction plots demonstrating the effects of night half (first and second halves of each sleep period) and night (before or after PBMT intervention) on (**A**) Minimum HR (bpm). (**B**) Maximum HR (bpm). (**C**) Average HR (bpm). (**D**) Average rate of change in HR per hour (bpm/hour). (*) denotes significant pairwise differences between the first and second halves of the night before PBMT intervention, (§) denotes significant pairwise differences between first and second halves of the night after PBMT intervention, (‡) denotes significant pairwise differences between the night before and night after PBMT intervention during the first half of the night only, and (¥) denotes significant pairwise differences between the night before and night after PBMT during the second half of the night only. (bpm, beats per minute; half*night, interaction between half of night and night; HR, heart rate; ME, main effect).

Evaluation of HR trends by half via two-way ANOVA determined significant ME of half on minimum HR (F(1,267.3) = 29.15, *p* < 0.0001), maximal HR (F(1,275.6) = 61.74, *p* < 0.0001), average HR (F(1,263.2) = 127.11, *p* < 0.0001), and rate of HR reduction per hour (F(1,269.8) = 249.44, *p* < 0.0001). Significant ME of night existed for minimum HR (F(1,267.3) = 9.15, *p* = 0.0027) and average HR (F(1,263.2) = 13.57, *p* = 0.0003). A significant interaction between half*night existed only for maximal HR (F(1,275.6) = 5.55, *p* = 0.0192). These patterns in HR by half of night are exhibited in Figure 2A–D.

**Figure 3:**
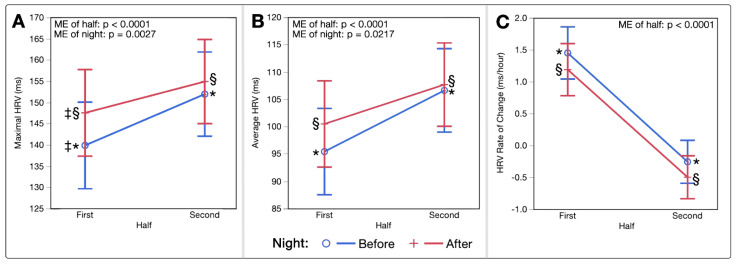
(**A**–**C**) Heart Rate Variability Trends by Half of Night. Least squares means interaction plots demonstrating the effects of night half (first and second halves of each sleep period) and night (before or after PBMT intervention) on (**A**) Maximum HRV (ms). (**B**) Average HRV (ms). (**C**) Average rate of change in HRV per hour (ms/hour). (*) denotes significant pairwise differences between the first and second halves of the night before the PBMT intervention, (§) denotes significant pairwise differences between first and second halves of the night after the PBMT intervention, and (‡) denotes significant pairwise differences between the night before and night after PBMT intervention during the first half of the night only. (HRV, heart rate variability; ME, main effect; ms, milliseconds).

Two-way ANOVAs assessing nightly HRV trends by half also resulted in significant ME of half on maximal HRV (F(1,268.6) = 30.89, *p* < 0.0001), average HRV (F(1,265.1) = 48.23, *p* < 0.0001), and rate of HRV increase per hour (F(1,273.6) = 68.51, *p* < 0.0001). Significant ME of night existed on maximal HRV (F(1,268.6) = 9.19, *p* = 0.0027) and average HRV (F(1,265.1) = 5.33, *p* = 0.0217). No significant interactions between half*night existed for HRV parameters. Figure 3A–C displays these patterns in HRV by half.

### 3.2. Training Load

**Figure 4:**
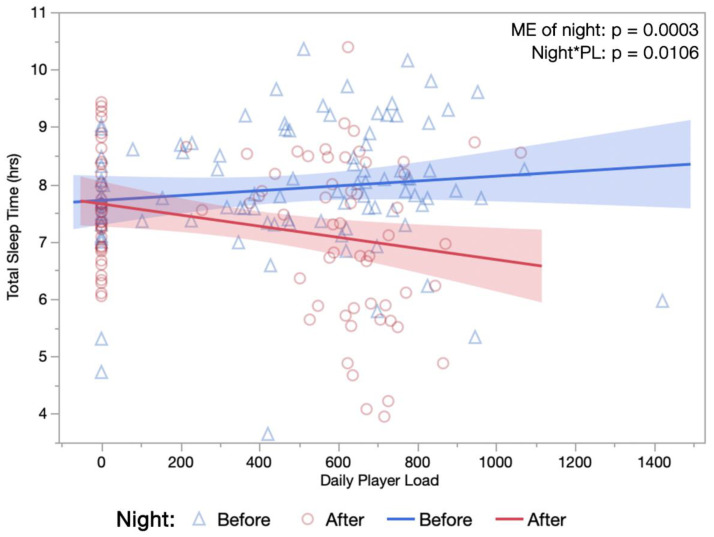
Relationship between Total Sleep Time and Daily Training Load. Single-day player loads (au) measured via GPS during training sessions are plotted against subsequent night sleep durations, occurring both the night before and night after a PBMT session. (hrs, hours; ME, main effect; night*PL, interaction between night and player load).

Table 4 lists fixed effect *p*-values from linear effect models evaluating single-day and cumulative, four-day player loads. Daily and cumulative player loads were found to have notable impacts on multiple physiological and sleep parameters, including TST; the significant ME of night (F(1,93.2) = 14.42, *p* = 0.0003) and interaction occurring between night*one-day player load (F(1,178.1) = 6.67, *p* = 0.0106) can be seen in Figure 4. 

## 4. Discussion

The present analysis is the first to observe free-living trends in sleep and cardiorespiratory physiology surrounding the use of full-body Photobiomodulation therapy (PBMT) in a cohort of female athletes within a NCAA “Power 5” conference. Athletes averaged 40 min less sleep the night after a PBMT session, while also demonstrating reductions in HR and trending increases in HRV, as compared to the night before the session. Collectively, higher physical training loads recorded during training had a tendency to increase HR and RR, independent of PBMT; however, changes in sleep quantity and composition demonstrated stronger associations with timing of the intervention, independent of daily or cumulative training loads. 

Previous research has shown HR to decrease throughout the night, most substantially during the high proportions of deep sleep in the first half, with a plateau sometimes reached prior to waking [49,50]. Opposing patterns are often seen in HRV, with values and their variability becoming greater with longer periods of REM sleep [37,38,50]. In the present analysis, longer sleep durations were associated with lower HR, higher HRV, and lower RR (Figure 1A–C); this is consistent independent of whether the sleep occurred before or after a PBMT session. Following PBMT, this relationship shifted to shorter sleep durations associated with a given HR, HRV, and RR. Interestingly, values across the first and second half of the night for HR and HRV followed the same relative patterns, although slight improvements existed in both halves post-intervention (Figure 2A–D and Figure 3A–C), keeping in mind that each half was on average 20 min shorter in duration post-intervention. 

With consideration to external training load, single-day and cumulative player loads corresponded positively with higher HR and RR, both before and after PBMT. Conversely, findings demonstrated that the PBMT intervention had a substantial influence on sleep, with decreases in TST, light duration, REM duration, and REM proportion following treatment, often unaffected by training loads. As demonstrated previously, one would expect higher training loads to be associated with longer sleep durations [51,52] and higher nocturnal HRs [44,53] as the body responds to greater physical stress; however, findings herein opposed these expected outcomes, with greater reductions in duration of total sleep and light sleep occurring after PBMT sessions when completed on days with higher single-day loads (Figure 4 and Table 4). These findings again demonstrate the same cardiorespiratory responses following exercise of varying intensity, through reductions in the amount of corresponding sleep obtained.

Potential mechanisms justifying the relationship between sleep and PBMT have not yet been suggested in the literature. Given that most sleep research has only demonstrated the consequences of failing to obtain sufficient sleep, as well as what may inhibit it, there is a scarce understanding of what may cause a reduction in required sleep. Augmented waste clearance from PBMT, which has been demonstrated previously [54,55], could enhance physiological efficiency and may justify the changes observed in sleep architecture. Both PBMT and sleep have been suggested to have implications in similar damage-repairing mechanisms; thus, it is possible that participation in full-body PBMT reduces the degree of restoration required during a subsequent sleep period, though this has not been evaluated. The present analysis did not consider post-PBMT physical or cognitive performance, nor immune function, albeit extensive prior research suggests PBMT results in enhancements across each domain [16,56,57,58]. While it cannot be ascertained that sleep herein was not inhibited, augmentations in HR and HRV patterns do not suggest a consequence on autonomic profile.

Sleep was monitored herein using the OURA Ring; the finger-worn ring estimates sleep related parameters via motion collected by 3D accelerometers and cardiorespiratory variables captured via photoplethysmography (PPG) [59]. In prior assessments, the OURA ring was found to quantify cardiac physiology and sleep durations with high degrees of accuracy [60,61,62,63,64]. It should be noted, however, that higher error rates have been found for the validation of sleep stage classification [60,63]. It should also be noted that sleep stages are estimated via relative changes in movement and cardiorespiratory parameters (HR, HRV and RR). The literature does not support whether PBMT could alter normal second-by-second relationships for these variables, which would ultimately impact algorithmic stage classification during sleep. Thus, further evaluation of sleep stages and cortical activity via EEG should be a focus of future research. 

Findings presented herein are novel to existing literature in that data was obtained in a free-living manner, rather than within laboratory conditions; this emphasizes the real-world translation of findings, but also presents with limitations. Sleep is a vulnerable behavior that is susceptible to changes by influence of exercise, nutrition, stress, and psychology, just to name a few [46]. While it is of high value to understand the relationships that each of these factors can hold with PBMT, it is also vital that evaluations are made under conditions that allow for high variability within each confound, similar to that of a real-world setting. As such, the possibility of a placebo effect cannot be ruled out from this observational analysis, as with any clinical intervention. Though, it should be noted that subjects were unaware of the hypothesis discussed herein at the time of collection, as data was not collected specifically for research purposes.

Extensive future research should be focused towards replicating these observations in the laboratory, as well as assessing performance longitudinally to ensure that these findings do not exist in combination with corresponding factors (e.g., reductions in psychomotor vigilance). Further, additional EEG-based assessments are needed for affirmation of these findings along with associated biological variables that may occur in combination, such as levels of metabolic waste, proinflammatory markers, and blood perfusion, to name a few.

In summary, casual integration of full-body PBMT by elite athletes was associated with observed single-night reductions in sleep duration concurrent with augmentations in autonomic profile (HR & HRV). Sleep is an active process involving most of the body’s systems that contributes to recovery through the maintenance of homeostasis [37,38]. A similar integrated perspective should be considered when using full-body PBMT to promote recovery. The involvement of numerous bodily systems in the recovery process suggests added utility for full-body PBMT as compared to the localized administration methods utilized throughout much of the literature, which tends to focus only on the obvious source of damage (i.e., key muscle groups). The findings presented herein provide a novel approach to determining the efficacy of PBMT that warrant continued research.

## Figures and Tables

**Table 1:** Dose and Beam Parameters.

Parameter	Visible Red	NIR
Wavelength	660 ± 25 nm	850 ± 30 nm
LED Quantity	1200	1200
Power per LED	0.267 W	0.267 W
Irradiance (at patient skin)	0.012 W/cm^2^	0.012 W/cm^2^
Total Emitted Power	321 W	321 W
Treatment Time	1200 s	1200 s
Energy Emitted	385,056 J	385,056 J
Fluence	14.4 J/cm^2^	14.4 J/cm^2^

Dosage and beam parameters for each completed session were provided by the device manufacturer, NovoTHOR. (cm, centimeters; FWHM, full width half-maximum; J, joules; NIR, near infra-red; nm, nanometers; s, seconds; W, watts).

**Table 2:** Pre-intervention vs. Post-intervention Nocturnal Parameters.

	Variable	Pre	Post	Mean Difference	*p*-Value	*d*
*Physiology*	Average HR (bpm)	54.31	53.31	0.996 (0.300, 1.691)	**0.0055** *	0.175
	Average HRV (ms)	100.46	103.73	−3.269 (−6.899, 0.362)	0.0770	0.251
	Average RR (rpm)	16.85	16.80	0.058 (−0.094, 0.210)	0.4504	0.022
*Sleep*	Total Sleep Time (h)	7.93	7.29	0.635 (0.282, 0.989)	**0.0006** *	0.156
	Awake Time (h)	1.35	1.28	0.070 (−0.061, 0.200)	0.2935	0.028
	Sleep Efficiency (%)	85.65	85.13	0.516 (−0.602, 1.634)	0.3616	0.071
	Light Duration (h)	4.62	4.30	0.322 (0.031, 0.613)	**0.0307** *	0.087
	Deep Duration (h)	2.24	2.12	0.114 (−0.030, 0.258)	0.1188	0.044
	REM Duration (h)	1.07	0.87	0.200 (0.075, 0.324)	**0.0019** *	0.083
	% Light	57.84	58.70	−0.862 (−3.178, 1.453)	0.4614	0.083
	% Deep	28.97	29.90	−0.933 (−3.109, 1.243)	0.3966	0.092
	% REM	13.21	11.41	1.798 (0.41, 3.185)	**0.0117** *	0.223

Mean difference expressed as Mean (lower 95% CI, upper 95% CI). Variables determined to be significantly different (α < 0.05) between pre-intervention and post-intervention by a paired *t*-test are in bold and denoted by an asterisk (*). (bpm, beats per minute; HR, heart rate; h, hours; HRV, heart rate variability; ms, milliseconds; pre, preintervention; post, postintervention; rpm, respirations per minute; REM, rapid eye movement; RR, respiration rate).

**Table 3:** Fixed Effects of Sleep on Cardiorespiratory Physiology.

Outcome Measure	Fixed Effect	F	df	*p*-Value
Average HR	Night	8.18	1, 90.7	0.0052 *
	TST	0.41	1, 102.4	0.5254
	Night*TST	0.59	1, 102.7	0.4451
Average HRV	Night	6.43	1, 89.0	0.0130 *
	TST	6.09	1, 98.5	0.0153 *
	Night*TST	3.05	1, 98.8	0.0839
Average RR	Night	1.05	1, 88.9	0.3090
	TST	0.86	1, 93.0	0.3552
	Night*TST	0.01	1, 93.1	0.9051

Linear mixed model fixed effect statistics demonstrating the relationship between sleep duration (TST) and night (pre-intervention vs. post-intervention) on cardiorespiratory parameters. Fixed effects determined to have a significant effect on the specified cardiorespiratory variable (α < 0.05) are in bold and denoted with an asterisk (*). (HR, heart rate; HRV, heart rate variability; night*TST, interaction between night and total sleep time; RR, respiration rate; TST, total sleep time).

**Table 4:** Effect of Daily and Cumulative Training Loads on Sleep and Nocturnal Physiology.

		Single-Day PL			4D Cumulative PL		
	Variable	Night	PL	Night*PL	Night	PL	Night*PL
*Physiology*	Average HR (bpm)	0.0643	<0.0001 *	0.0761	0.0089 *	0.0132 *	0.6472
	Average HRV (ms)	0.1598	0.1793	0.1916	0.0912	0.5113	0.6187
	Average RR (rpm)	0.8956	0.0004 *	0.0549	0.6076	0.0140 *	0.6390
*Sleep*	Total Sleep Time (h)	0.0003 *	0.3309	0.0106 *	0.0005 *	0.1993	0.8867
	Awake Time (h)	0.2438	0.4530	0.9029	0.2798	0.4659	0.9330
	Sleep Efficiency (%)	0.4060	0.7732	0.2514	0.3742	0.8003	0.8906
	Light Duration (h)	0.0299 *	0.8949	0.0401 *	0.0301 *	0.7022	0.7982
	Deep Duration (h)	0.0881	0.3869	0.1862	0.1087	0.3192	0.5547
	REM Duration (h)	0.0016 *	0.6175	0.2155	0.0018 *	0.3259	0.7460
	% Light	0.4304	0.7136	0.9402	0.4461	0.5736	0.4394
	% Deep	0.4016	0.9552	0.4968	0.3929	0.8361	0.7182
	% REM	0.0098 *	0.6314	0.2001	0.010 5 *	0.3244	0.4464

Fixed effect *p*-values for linear mixed effect models evaluating the relationship that night and physical training loads have with physiological and sleep parameters. Each variable was evaluated for main effect of player load (PL, separately for single-day and cumulative four-day load), main effect of night (pre-intervention vs. post-intervention), and interactions between player load and night. Fixed effects determined to have a significant effect on the specified physiological variable (α < 0.05) are in bold and denoted with an asterisk (*). (bpm, beats per minute; HR, heart rate; h, hours; HRV, heart rate variability; ms, milliseconds; night*PL, interaction between night and player load; PL, player load; REM, rapid eye movement; rpm, respirations per minute; RR, respiration rate; 4D, four day).

## Data Availability

The data underlying this article cannot be shared publicly due to certain elements of the data being owned by a third party and are considered confidential. The data will be shared on reasonable request to the corresponding author.
